# Prostaglandin contribution to postexercise hyperemia is dependent on tissue oxygenation during rhythmic and isometric contractions

**DOI:** 10.14814/phy2.14471

**Published:** 2020-06-19

**Authors:** Rehan T. Junejo, Clare J. Ray, Janice M. Marshall

**Affiliations:** ^1^ School of Sport, Exercise & Rehabilitation Sciences College of Life & Environmental Sciences Birmingham UK; ^2^ Institute of Clinical Sciences College of Medical and Dental Sciences University of Birmingham Birmingham UK

**Keywords:** exercise‐hyperemia, oxygen‐dependent, prostaglandins

## Abstract

The role of prostaglandins (PGs) in exercise hyperemia is controversial. We tested their contributions in moderate intensity forearm exercise, whether their release is oxygen (O_2_)‐dependent or affected by aging. A total of 12 young (21 ± 1 years) and 11 older (66 ± 2 years) recreationally active men performed rhythmic and isometric handgrip contractions at 60% maximum voluntary contraction for 3 min during air breathing after placebo, after cyclooxygenase (COX) inhibition with aspirin, while breathing 40% O_2_ and during their combination (aspirin + 40% O_2_). Forearm blood flow (FBF) was recorded with venous occlusion plethysmography (forearm vascular conductance (FVC): FBF/mean arterial pressure). Venous efflux of PGI_2_ and PGE_2_ were assessed by immunoassay. Postcontraction increases in FVC were similar for rhythmic and isometric contractions in young and older men, and accompanied by similar increases in efflux of PGI_2_ and PGE_2_. Aspirin attenuated the efflux of PGI_2_ by 75%–85%, PGE_2_ by 50%–70%, (*p* < .05 within group; *p* > .05 young versus. older), and postcontraction increases in FVC by 22%–27% and 17%–21% in young and older men, respectively (*p* < .05 within group and young versus. older). In both age groups, 40% O_2_ and aspirin + 40% O_2_ caused similar inhibition of the increases in FVC and efflux of PGs as aspirin alone (*p* < .05 within group). These results indicate that PGs make substantial contributions to the postcontraction hyperemia of rhythmic *and* isometric contractions at moderate intensities in recreationally active young *and* older men. Given PGI_2_ is mainly released by endothelium and PGE_2_ by muscle fibers, we propose PG generation is dependent on the contraction‐induced falls in O_2_ at these sites.

## Graphical abstract

There is controversy over whether prostaglandins contribute to exercise hyperemia, especially with aging. We show that dilator prostaglandins are responsible for 17%–27% of exercise hyperemia following medium intensity rhythmic or static contractions in both young and older, recreationally active men, as judged by the effects of cyclooxygenase inhibition on muscle blood flow and prostaglandin efflux. The contributions of prostaglandins are attenuated by breathing 40% oxygen indicating prostaglandin synthesis is triggered by a fall in muscle oxygenation.

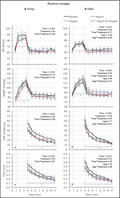

## 1 INTRODUCTION

There is substantial evidence that prostaglandins (PGs) contribute to exercise hyperemia, but there is also conflicting evidence. For example inhibition of cyclooxygenase (COX) attenuated postcontraction hyperemia associated with rhythmic and isometric contractions of forearm and leg (Cowley, Stainer, Rowley, & Wilcox, 1985; Duffy, New, Tran, Harper, & Meredith, 1999; Kilbom & Wennmalm, 1976; Win & Marshall, 2005). However, it was separately reported that COX inhibition had no effect on hyperemia during rhythmic contraction in forearm (Shoemaker, Naylor, Pozeg, and Hughson (1996); Mortensen, González‐Alonso, Damsgaard, Saltin, & Hellsten, 2007), and that combined inhibition of COX *and* nitric oxide (NO) synthase (NOS) was required to attenuate the hyperemia (Boushel et al., 2002; Mortensen et al., 2007). These findings led to the suggestion that PGs and NO contribute synergistically, rather than independently, to exercise hyperemia (Boushel et al., 2002; Mortensen et al., 2007). In contrast, the observation that the attenuating effect of COX inhibition on hyperemia during rhythmic contraction was transient, whereas that of NOS inhibition was sustained led to the proposal that the contribution of PGs to exercise hyperemia is independent of NO, and can be compensated for by other dilator/s (Schrage, Joyner, & Dinenno, 2004).

A possible explanation for these disparities is that they reflect differences between studies in exercise intensity and a possible fall in partial pressure of O_2_ (PO_2_) within muscles. For, those which suggested a relatively minor contribution of PGs to exercise hyperemia used exercise intensities of ≤20% maximum (Mortensen et al., 2007; Schrage et al., 2004; Shoemaker et al., 1996), whereas those suggesting a substantial contribution used intensities of ≥60% maximum (Kilbom & Wennmalm, 1976; Win & Marshall, 2005). In line with this idea, PGE_2_ release into muscle interstitium during isometric contraction was enhanced by arterial occlusion, which would have greatly reduced tissue PO_2_ (Symons, Theodossy, Longhurst, & Stebbins, 1991). Moreover, the release of PGI_2_ into venous efflux and PGI_2_ and PGE_2_ into muscle interstitium during rhythmic exercise was directly related to O_2_ consumption (VO_2_) and exercise intensity (Karamouzis, Karamouzis, & Vamvakoudis, 2001; Zoladz, Majerczak, Duda, & Chlopicki, 2009). Furthermore, the postcontraction hyperemia of isometric handgrip contraction at 60% maximum voluntary contraction (MVC) was similarly attenuated by breathing 40% O_2_ or COX inhibition, whereas combined COX inhibition and 40% O_2_ had no greater effect (Win & Marshall, 2005). Also, when breathing 40% O_2_ was restricted to the period of isometric contraction, postcontraction hyperemia was attenuated, whereas 40% O_2_ from contraction cessation had no such effect (Fordy & Marshall, 2012). Thus, we proposed 40% O_2_ alleviates the fall in tissue PO_2_ decreasing the generation of PO_2_‐dependent PGs by endothelium and/or skeletal muscle (Fordy & Marshall, 2012; Frisbee, Maier, Falck, Roman, & Lombard, 2002; Marshall & Ray, 2012; Michiels, Arnould, Knott, Dieu, & Remacle, 1993; Win & Marshall, 2005). However, uncertainty remains over this interpretation because the higher PO_2_ attained with 40% O_2_ may prevent the *action,* rather than release of PGs. Furthermore, as muscle blood flow is limited persistently during isometric, but intermittently during rhythmic contractions (Kagaya & Homma, 1997; McNeil, Allen, Olympico, Shoemaker, & Rice, 2015; Van Beekvelt, Shoemaker, Tschakovsky, Hopman, & Hughson, 2001), the fall in tissue PO_2_ during isometric contraction may have greater effects on PG synthesis.

Separately, there is also uncertainty over the effects of aging on the contribution of PGs to exercise hyperemia. In contrast to young subjects ( Schrage et al., 2004), COX inhibition had no effect on hyperemia during 10% MVC rhythmic contractions in older subjects, leading the authors to conclude that the role of PGs is lost with aging (Schrage, Eisenach, & Joyner, 2007). Furthermore, forearm vasodilator responses to infused PGI_2_ were smaller in older than young subjects (Nicholson, Vaa, Hesse, Eisenach, & Joyner, 2009). However, the older subjects who took part in those studies were relatively inactive (Nicholson et al., 2009; Schrage et al., 2007). Although muscle VO_2_ is maintained during submaximal exercise in both recreationally active and sedentary older men, exercise hyperemia was only blunted in latter (Poole, Lawrenson, Kim, Brown, & Richardson, 2003; Proctor et al., 2003). Thus, the loss of PG involvement in exercise hyperemia with aging (Schrage et al., 2007) may have reflected aging, sedentariness, the light intensity rhythmic exercise and small fall in muscle PO_2_ (Van Beekvelt et al., 2001), and/or impaired responsiveness to PGs (Nicholson et al., 2009; Schrage et al., 2007).

With this background, we hypothesized that in recreationally active young and older men, rhythmic and isometric contractions at moderate intensity of 60% MVC would increase venous efflux of both PGI_2_ and PGE_2_, but their efflux would be greater in isometric contraction and greater in young men. Furthermore, breathing 40% O_2_ or COX inhibition would similarly attenuate postcontraction hyperemia and PG efflux following rhythmic *and* isometric contractions in both young *and* older men. We focussed on men to avoid the complicating facilitatory influences of estrogen on COX and NOS activity (Orshal & Khalil, 2004). Some of these results have been published in brief (Junejo, Ray, & Marshall, 2014, 2015).

## 2 METHODS

This study was approved by the University of Birmingham's Ethnical Review Committee (Project ERN_12‐1377**)** and undertaken in accordance with the revisions of *Declaration of Helsinki*.

### 2.1 Subjects

A total of 12 young and 11 older men (age 21 ± 1 and 66 ± 2 years, respectively) who were students or staff of the University of Birmingham, or members of The Birmingham 1000 Elders Group were recruited for the study. All regularly participated in recreational activities, but none were in training. None took prescribed medication. Prior to the experimental session, participants were requested to refrain from caffeinated drinks and heavy meals for ≥12 hr; alcohol consumption, nonsteroidal anti‐inflammatory drugs, or strenuous exercise for ≥24 hr.

### 2.2 Experimental procedures

During a familiarization visit, written informed consent was obtained following explanation of the protocol. MVC of the dominant hand was recorded using a handgrip dynamometer (Lafayette 70718, Loughborough, UK) as an average of 3 maximal effort 5 s handgrip contractions separated by at least 30 s. Habituation to experimental conditions was aided by practising the protocol during this session.

For each experimental session, the subject rested supine on a couch with the backrest at ~65° and both arms supported at heart level. An intravenous cannula (22–24 G, BD Venflon, BD and Co.) was inserted in the antecubital vein of the dominant (exercising) forearm to allow blood sampling. Beat by beat arterial blood pressure (ABP) was monitored from a finger on the nondominant hand using photoplethysmography (Finapres, Ohmeda 2300, Englewood, USA); Mean ABP (MABP), and heart rate (HR) were computed from the pulsatile ABP trace. The dominant arm was arranged so that handgrip contraction could be performed with the dynamometer; a visual display and audible metronome allowed maintenance of the requested contractions. Forearm blood flow (FBF) was recorded from the exercising arm using an electrically calibrating venous occlusion plethysmograph (EC6 Plethysmograph with E20 rapid cuff inflator, D.E. Hokhansen Inc.). An indium‐gallium silastic strain‐gage was mounted on the widest part of the forearm. For each FBF measurement, a venous collecting cuff wrapped around the upper arm was inflated to 50 mmHg; a second cuff around the wrist was inflated to >250 mmHg ~6 s before inflation of the upper arm cuff. FBF was calculated from the slope of the strain‐gage output over the 1st complete pulsatile cardiac‐cycle beat, in accordance with published guidelines (Junejo, Ray, & Marshall, 2019). Forearm vascular conductance (FVC) was calculated as FBF divided by MABP recorded over the same cardiac‐cycle/s and expressed as conductance units (CU).

### 2.3 Experimental protocol

The protocol was carried out on four different days in a randomized, single‐blind, cross‐over design under control conditions (placebo/air breathing) and with three treatments: aspirin, 40% O_2_, and the combination; aspirin + 40% O_2_. On arrival, the subject consumed an orange‐flavoured drink either without (placebo), or with aspirin (600 mg), which produces its maximal inhibition of COX at ~30 min (Heavey, Barrow, Hickling, & Ritter, 1985). The recording equipment and venous cannula were put in place and an equilibration period of ~20 min was allowed; the cannula was kept patent by infusion of 3 ml sterile saline bolus immediately after insertion and 15–20 s prior to each blood sampling (PosiFlush SP Syringe 0.9% NaCl, BD and Co.). Subject then breathed either medical grade air or 40% O_2_ via the facemask (100% O_2_ titrated using a Venturi valve); an O_2_ sensor (ProOx 110, BioSpherix) ensured appropriate delivery of the required O_2_ concentrations. After 5 min, resting venous samples were taken and baseline measurements of FBF were recorded and at 30 min after placebo/aspirin drink, the subject performed rhythmic handgrip contractions at 60% MVC (1 s contraction: 1 s relaxation) for 3 min. FBF was recorded immediately the final contraction ceased (0 s), at 30 s, 1 min and at 1 min intervals until 7 min. Values of MABP and HR were extracted for analysis over the same time periods as the FBF measurements and at the mid‐point of the 1st, 2nd, and 3rd min of contractions. In all subjects, venous blood samples were taken for blood gas and metabolite analysis (see below) at rest, immediately the contractions ceased (0 s), 3‐, 5‐, and 7‐min postexercise. Furthermore, in six randomly selected subjects from each age group, additional blood samples were taken at rest and immediately the contractions ceased for assay of PG metabolites (see below). The subject then rested whilst breathing normal room air for 25 min.

This protocol was repeated except the subject performed 60% MVC isometric handgrip contraction for 3 min, or as long as possible, with vigorous verbal encouragement. Care was taken to ensure the subject did not engage in a Valsalva manoeuvre. Blood samples were taken as described for rhythmic contractions.

### 2.4 Blood analysis

Venous blood samples were collected into a 1 ml syringe for immediate analysis of pO_2_, pCO_2_, pH, K^+^, and lactate (GEM Premier 4000, Instrumentation Laboratory). Samples for PG assay were collected into ice‐cold heparinized vacutainers with 1 µl/ml of 10 µM indomethacin (Sigma Aldrich). Vacutainers were centrifuged at 700 *g* at 4°C for 20 min (Mistral 3000i, MSE Ltd). Supernatant plasma was collected in 1 ml Eppendorf tubes, snap frozen in liquid nitrogen and stored in −80°C. Enzyme‐linked immune‐sorbent assays (ELISAs) were performed for PGI_2_ and PGE_2_ derivatives [6‐Keto PGF_1α_ and PGE_2_ metabolite (PGEM)] using commercially available kits (Cayman Chemical Co.). The efflux of each PG was calculated for each subject at baseline and on cessation of exercise as the product of venous PG concentration and the associated FBF value.

### 2.5 Data Analysis

Hemodynamic and handgrip contraction data were digitally collected at 400 Hz using Power‐Lab and Lab Chart data acquisition software (Version 7.3.3 AD Instruments) and stored on a desktop computer (Dell Inc.). Data were analysed on JMP for Windows (Version 13.0.0, SAS Institute Inc.). Tension time integral (TTI) from the dynamometer output, relative percentage change in PG efflux and in postcontraction hyperemia (FVC values from 0 s until 7 min after contraction) were computed offline. Participant characteristics were compared between the age groups using Student's *t*‐test. Factorial mixed‐model analysis of variance (ANOVA) was used to identify time, treatment, age, and interaction effects. Additionally, time effect within each treatment were analyzed using one‐way repeated‐measures ANOVA. Once a significant effect was detected, Tukey's HSD was used as post hoc test. Statistical significance was set at *p* < .05.

## 3 RESULTS

### 3.1 Subject characteristics

Both groups were closely matched for height, weight, body mass index, and forearm circumference; cardiovascular baselines were also similar. However, as expected, age and MVC were different between the two groups. These variables along with all nutritional supplementation use are shown Table 1.

**TABLE 1:** Participant characteristics of young and older men

	Young	Older	*p* value
*n*	12	11	—
Age (year)	21 ± 1	66 ± 2	<.001
Height (m)	1.78 ± 0.02	1.76 ± 0.02	.65
Weight (Kg)	74.9 ± 3.1	77.3 ± 0.8	.18
Body mass index (Kg/m^2^)	23.6 ± 0.5	24.8 ± 0.8	.18
Forearm circumference (cm)	26.3 ± 0.6	25.0 ± 1.0	.14
MVC (*N*)	229.3 ± 24.4	172.1 ± 10.9	.049
Nutritional supplements (*n*)			
Creatine	3	—	—
Glutamine	3	—	—
Multivitamins	3	1	—
Fish oil	2	1	—
Rhythmic handgrip			
HR (b/min)	64 ± 1	65 ± 2	.85
MABP (mmHg)	79 ± 3	84 ± 2	.45
FBF (ml dl^−1^ min^−1^)	4.45 ± 0.43	6.59 ± 0.77	.17
FVC (CU)	0.06 ± 0.01	0.08 ± 0.01	.28
Isometric handgrip			
HR (b/min)	68 ± 2	62 ± 2	.08
MABP (mmHg)	80 ± 3	82 ± 2	.83
FBF (ml dl^−1^ min^−1^)	5.92 ± 0.55	6.67 ± 0.67	.60
FVC (CU)	0.08 ± 0.01	0.09 ± 0.01	.69

Note

Values are mean ± *SE*. Abbreviations: FBF, horearm blood flow; FVC, forearm vascular conductance; HR, heart rate; MABP, mean arterial blood pressure; MVC, maximum voluntary contraction. *p* values: young versus older.

### 3.2 Magnitude of forearm contractions

The older men achieved a MVC that was ~25% less than that of the young men (*p* < .05; Table 1). Within each age group, the TTI for rhythmic handgrip was lower than for isometric handgrip (Table 2). The TTIs for rhythmic and isometric contractions were not significantly different between older and young men, but fewer young men completed the full 3 min of isometric handgrip. None of the treatments (aspirin, 40% O_2_, or aspirin + 40% O_2_) affected TTI for rhythmic or isometric contraction in young, or older men.

**TABLE 2:** Tension Time Index (TTI) and duration of rhythmic and isometric contractions in Young and Older men

Young	Placebo	Aspirin	40% O_2_	Aspirin/40% O_2_	*p* value
TTI (KN.s)					
Rhythmic	10.6 ± 1.4	10.6 ± 1.2	10.8 ± 1.4	10.8 ± 1.4	.99
Isometric	16.9 ± 3.1	17.2 ± 2.1	16.4 ± 2.5	16.2 ± 2.6	.85
*P* value	Exercise type < 0.001; exercise type*treatment = 0.87				
Older					
Time (min)					
Rhythmic	3.0 ± 0.0	3.0 ± 0.0	3.0 ± 0.0	3.0 ± 0.0	.49
Isometric	2.3 ± 0.1	2.6 ± 0.1	2.3 ± 0.1	2.3 ± 0.2	.35
*p* value	Exercise type < 0.001; exercise type * treatment = 0.35				
TTI (KN.s)					
Rhythmic	8.3 ± 0.7	8.1 ± 0.8	7.8 ± 0.7	8.2 ± 0.7	.97
Isometric	15.6 ± 1.4	16.2 ± 1.3	15.4 ± 1.4	15.9 ± 1.3	.98
*p* value	Exercise type < 0.001; exercise type*treatment = 0.99; Age = **0 0.13**; Age*exercise type = **0.21**				
Time (min)					
Rhythmic	3.0 ± 0.0	3.0 ± 0.0	3.0 ± 0.0	3.0 ± 0.0	.85
Isometric	3.0 ± 0.0	3.0 ± 0.0	3.0 ± 0.0	3.0 ± 0.0	.45
*p* value	Exercise type = 0.67; exercise type*treatment = 0.40; Age **< 0.001**; Age*exercise type **< 0.001**				

Note

All values are shown as mean ± *SEM*. *p* values in final column indicate comparisons *within* rhythmic or isometric contraction under different conditions. *p* values in rows indicate comparisons *between* rhythmic and isometric contraction and interactions between type of contraction and treatment within age group. *p* values shown in bold indicate comparisons between age groups.

### 3.3 Hemodynamic responses

**FIGURE 1:**
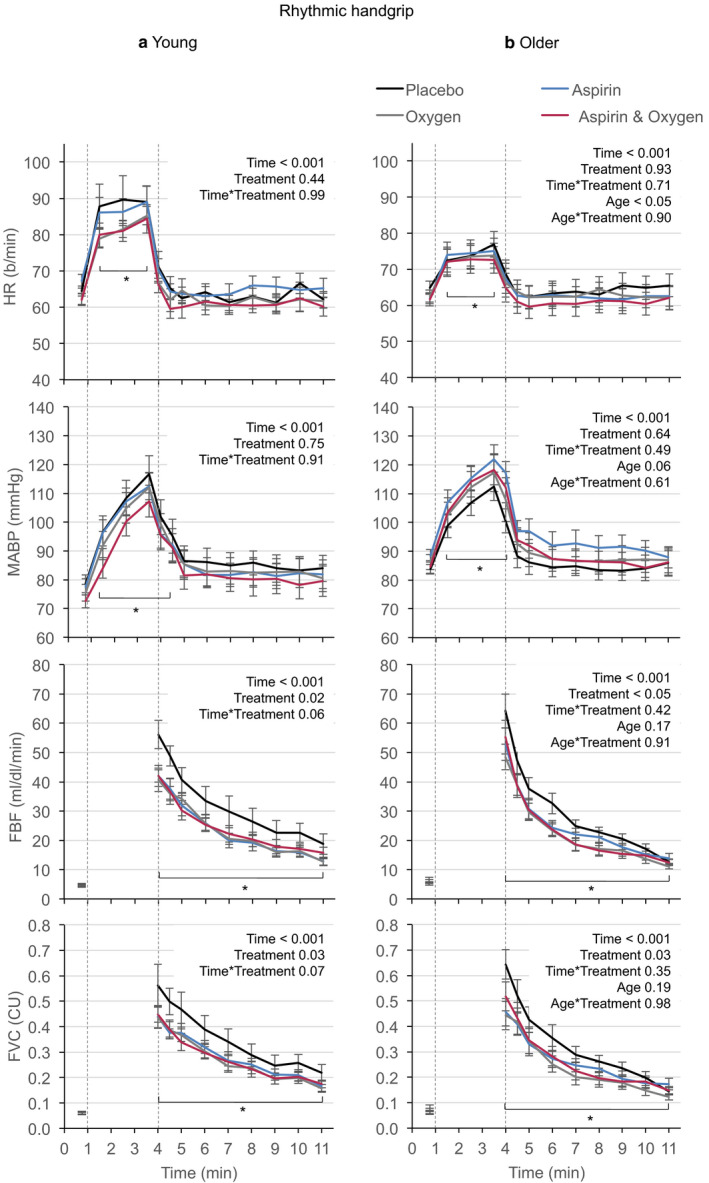
Changes in HR, MABP, FBF, and FVC evoked by rhythmic handgrip contractions performed by young (a) men and older (b) men under control conditions (placebo), after Aspirin, during 40% O_2_ and during combined Aspirin + 40% O_2_. Absolute values are shown as mean ± *SEM*. Dashed vertical lines show 3‐min period of contractions starting at 1 min and ending at 4 min. ABP and HR values were extracted during each min of contraction, and simultaneously with each measurement of FBF: baseline, immediately contraction ceased, at 30 s and at 1 min intervals until 7 min (FVC: FBF/ABP). *p < .05 all conditions versus their respective baselines

**FIGURE 2:**
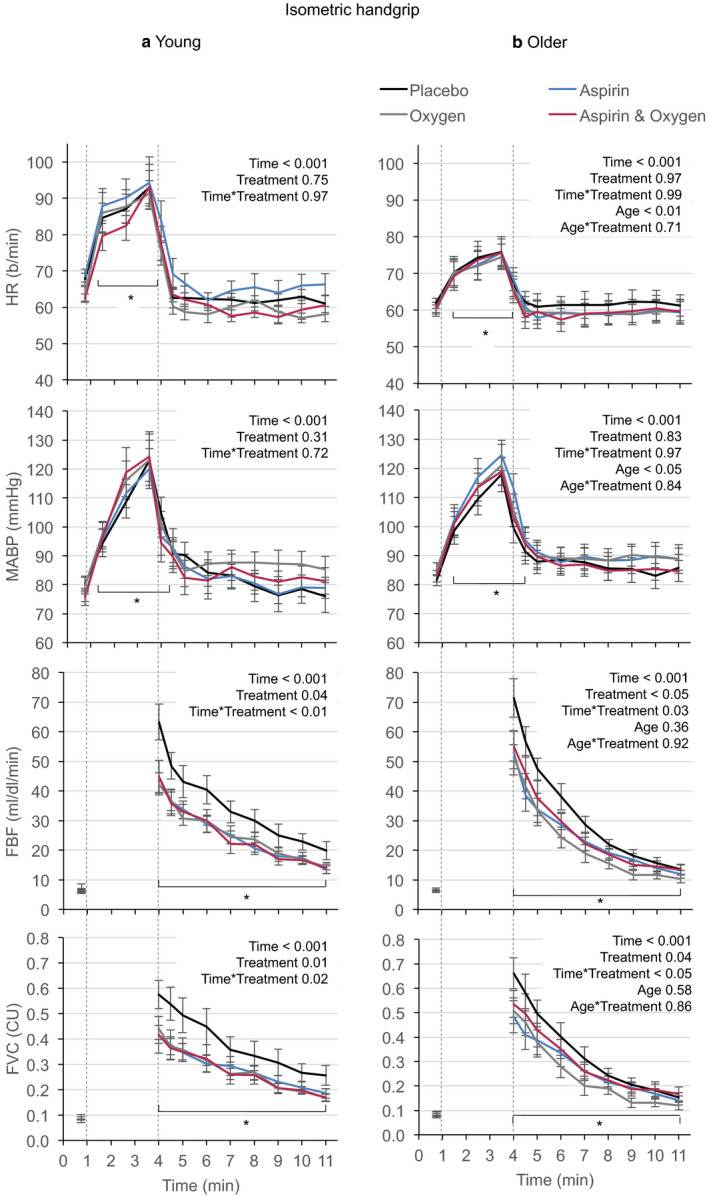
Changes in HR, MABP, FBF, and FVC evoked by isometric handgrip contractions performed by young (a) men and older (b) men under control conditions (placebo), after Aspirin, during 40% O_2_, and during combined Aspirin + 40% O_2_. Absolute values are mean ± *SEM*. Dashed vertical lines show 3‐min period of contractions starting at 1 min and ending at 4 min. ABP and HR values were extracted during each min of contraction, and simultaneously with each measurement of FBF: baseline, immediately contraction ceased, at 30 s and at 1 min intervals until 7 min (FVC: FBF/ABP). *p < .05 all conditions versus their respective baselines

Under control conditions, rhythmic and isometric contractions evoked smaller increases in HR in older men (*p* < .05; Figures 1 and 2), as described previously (Proctor et al., 2003) for submaximal exercise in recreationally active older men. None of the three treatments affected the increases in HR or MABP evoked by rhythmic or isometric handgrip contractions in young, or older men (Figures 1 and 2). However, all three treatments caused similar attenuation in absolute terms, of the postcontraction increases in FBF and FVC for rhythmic and isometric contraction in both young and older men (Figures 1 and 2).

### 3.4 Venous prostaglandins (PGs)

**FIGURE 3:**
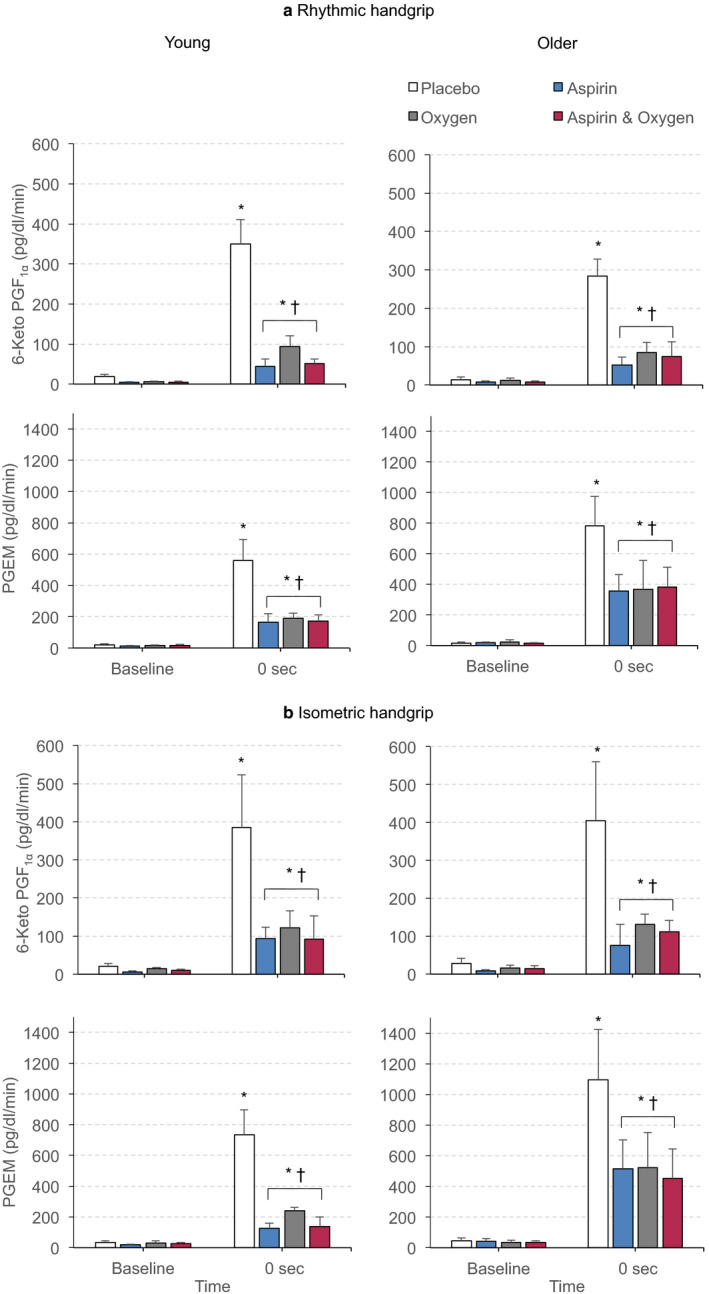
Increases in venous efflux of 6‐Keto PGF_1α_ and PGEM evoked in young men and older men by rhythmic (a) and isometric (b) contractions under control conditions (placebo), after Aspirin, during 40% O_2_, and during combined Aspirin + 40% O_2_. Absolute values are shown as mean ± *SEM* (*n* = 6 for each set of values) at baseline and immediately contractions ceased (time 0). *p < .05 versus respective baselines for all conditions included in bracket, ^†^ *p* < .05 for all treatments versus placebo

Under control conditions, baseline venous concentrations of 6‐Keto PGF_1α_ and PGEM did not differ between rhythmic and isometric handgrip contraction in either age group. There were also no differences between young and older men for baseline concentrations of 6‐Keto PGF_1α_ or PGEM (see online Figure S1, which shows the effects of the three treatments on venous PG concentrations before and following handgrip contractions). Considering the venous efflux data, under control conditions_,_ both rhythmic and isometric contractions led to significant increases in the venous efflux of 6‐Keto PGF_1α_ and PGEM in both young and older men (Figure 3). There were no differences between the age groups for venous efflux of either PG, but there was a strong trend for PGEM efflux to be greater in older men (Figure 3; *p* = .05 for isometric contractions). The effluxes of both PG metabolites caused by rhythmic and isometric contractions were substantially attenuated after aspirin in both young and older men (Figure 3). They were also reduced in both groups by 40% O_2_ and by combined aspirin + 40% O_2_ (Figure 3).

### 3.5 Venous PO_2_ (PvO_2_) and other metabolites

**FIGURE 4:**
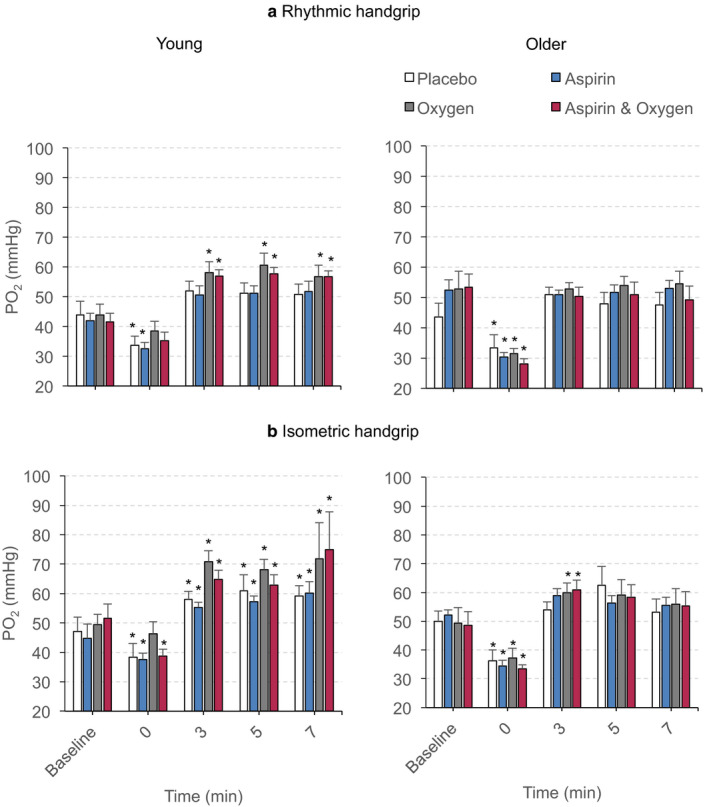
Changes in PvO_2_ evoked in young men and older men by rhythmic (a) and isometric (b) contractions under control conditions (placebo), after Aspirin, during 40% O_2_ and during combined Aspirin + 40% O_2_. Absolute values are shown as mean ± *SEM* at baseline, immediately contractions ceased (time 0) and at 3‐, 5‐, and 7‐min postcontractions. *p < .05 versus respective baselines

Overall, in young men, PvO_2_ values following both rhythmic and isometric handgrip contractions were significantly higher during 40% O_2_ and combined aspirin + 40% O_2_ than during control, or aspirin conditions (Treatment effects: *p* < .05 in each case, see Figure 4, Tables S1 and S2). Considering the time points in more detail, immediately following rhythmic and isometric contractions (i.e., at time 0), PvO_2_ values were *lower* than their respective baselines, except during 40% O_2_ and combined aspirin + 40% O_2_ for rhythmic contraction and during 40% O_2_ for isometric contraction (Figure 4). However, from 3 to 7 min, PvO_2_ values were *higher* than their respective baselines during 40% O_2_ and combined aspirin + 40%O_2_ following rhythmic contractions and under all conditions following isometric contractions (Figure 4).

In contrast, in older men, PvO_2_ values were not different between treatment conditions following rhythmic, or isometric contractions (Treatment effects: *p* = .91 and *p* = .99, respectively, see Figure 4). In fact, immediately following rhythmic and isometric contractions (at time 0), PvO_2_ values were lower than their respective baselines under all treatment conditions irrespective of whether 40% O_2_ was breathed (*p* < .01 vs. respective baselines). Furthermore, at 3–7 min following rhythmic and isometric contractions, PvO_2_ values were generally not significantly different from their respective baselines. It was only at 3 min following isometric contractions, that PvO_2_ reached values significantly higher than baselines during 40% O_2_ and combined aspirin + 40% O_2_ conditions (Figure 4). Tables S1 and S2 provide the numerical data for these changes.

As expected, venous PCO_2_ (PvCO_2_), K^+^, and lactate were increased, whereas venous pH was decreased immediately following both rhythmic and isometric contractions in both young and older men (Tables S1 and S2). Thereafter, PvCO_2_ and K^+^ returned to baseline levels by 3‐min post exercise, lactate was still raised at 7 min following both types of exercise, whereas pH tended to return to baseline more quickly in older, than young subjects. There were no treatment effects on the changes in PvCO_2_, K^+^, lactate, or pH. The only age‐dependent effects were on lactate and pH, which showed greater changes from baseline in young men (Tables S1 and S2).

### 3.6 Relative effects of aspirin, 40% O_2_ and aspirin/40% O_2_ in young and older subjects

**FIGURE 5:**
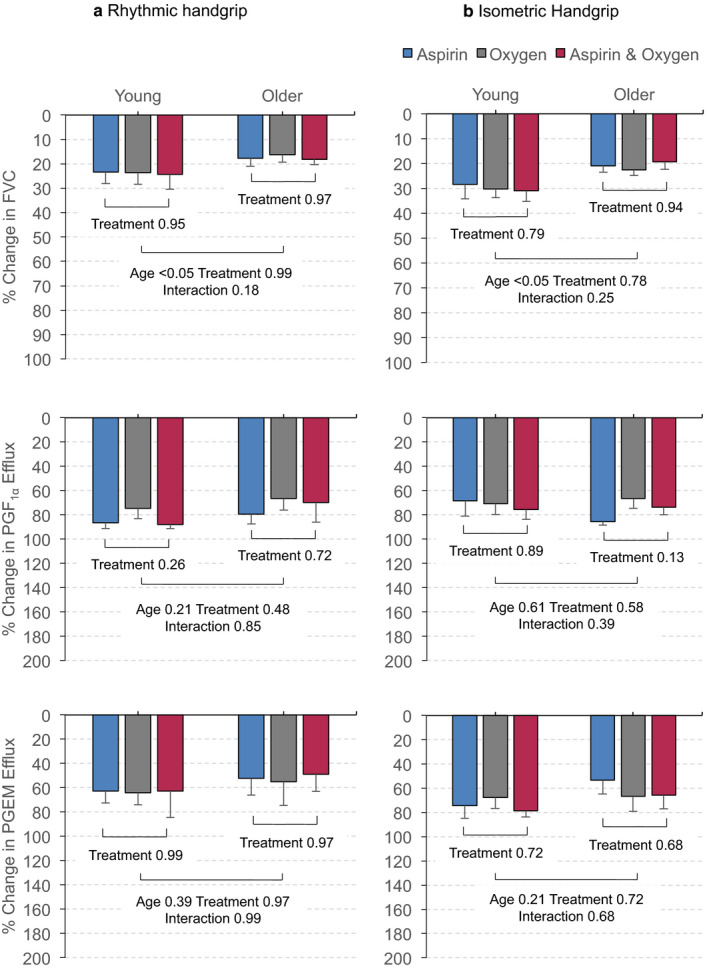
Percentage attenuation of peak postcontraction hyperemia and venous effluxes of 6‐Keto PGF_1α_ and PGEM following rhythmic (a) and isometric (b) contractions in young and older men. Values are percentage reductions in peak postcontraction FVC, and in effluxes of PGI_2_ and PGE_2_ metabolites caused by aspirin, 40% O_2_ and combined aspirin + 40% O_2_ shown as mean ± *SEM*. Each set of values relates to six young and six older subjects in whom PGs were assayed. *p* values for comparisons within and between rhythmic contractions in each age group and comparisons between age groups are shown below columns

Considering just the six young men in whom PG efflux was assayed, aspirin, 40% O_2_, and combined aspirin + 40% O_2_ attenuated the postcontraction vasodilatation (increase in FVC) of rhythmic and isometric handgrip contractions by ~24 and ~30%, respectively, relative to control responses, whereas in the six older men, the attenuations were significantly smaller: ~17 and ~21% for rhythmic and isometric contractions, respectively (see Figure 5; *p* < .05 vs. young). Table S3 provides the numerical data for these percentage changes.

In contrast, as shown in Figure 5 and Table S3, aspirin caused similar, substantial reductions in young and older men of contraction‐induced venous effluxes of 6‐Keto PGF_1α_, by 70%–85% and of PGEM, by 52%–75%. Furthermore, 40% O_2_ and combined aspirin + 40% O_2_ caused similar reductions as aspirin for both types of contraction in both young and older men. The relative reductions in effluxes of PG metabolites were not significantly different between young and older men.

## 4 DISCUSSION

This is the first study to make direct comparisons between recreationally active young and older men of the contributions made by newly generated PGs to postexercise hyperemia for isometric, or rhythmic contractions. Taken together, our findings indicate that in both young and older men, PGs make substantial O_2_‐dependent contributions to postexercise hyperemia evoked by contractions of moderate intensity, whether they are isometric, or rhythmic.

### 4.1 Control responses

Maximum voluntary contraction was smaller in older than young men, consistent with a loss of muscle mass and relative increase in the proportion of oxidative fibers with aging ( McGregor, Cameron‐Smith, & Poppitt, 2014). Nevertheless, in both young and older men, the TTI was greater for isometric, than rhythmic contractions, but postcontraction hyperemia was similar for the two types of contraction. This may be attributed to a greater metabolic cost of rhythmic contractions (Newham, Jones, Turner, & McIntyre, 1995), and greater release of vasodilator metabolites. Importantly, compared at the same relative workload (60% MVC), postcontraction vasodilator responses were similar in young and older men. Others made similar observations in recreationally active young and older subjects (Jasperse, Seals, & Callister, 1994; Proctor et al., 2003), whereas in sedentary older subjects, exercise hyperemia was blunted (Poole et al., 2003).

Under control conditions, the concentrations of PGI_2_ and PGE_2_ metabolites in plasma were, as expected, in the low pg/ml range in young and older men (Heavey et al., 1985; Trappe & Liu, 2013). In young men, the increases in venous efflux of both PGs following rhythmic contractions were consistent with previous assays of venous blood and muscle interstitial fluid (Boushel et al., 2002; Karamouzis et al., 2001; Wilson & Kapoor, 1993). We now show that venous effluxes of PGI_2_ and PGE_2_ are also increased following isometric contraction. Moreover, we report the novel finding that venous effluxes of PGI_2_ and PGE_2_ are increased in *older* men following both rhythmic and isometric contraction at 60% MVC.

### 4.2 Effects of COX inhibition

COX inhibition (aspirin: 600 mg p.o.) reduced efflux of both PGs at rest, and attenuated contraction‐induced efflux of PGI_2_ by 70%–85% in young *and* older men, consistent with evidence that the same dose of aspirin inhibited bradykinin‐induced generation of PGI_2_ by 85% and ~70% at 30 and 90 min, respectively ( Heavey et al., 1985). That PGE_2_ efflux was also inhibited by 50%–75% accords with evidence that oral aspirin attenuates the increase in interstitial PGE_2_ evoked by rhythmic contractions (Trappe & Liu, 2013).

By infusing COX inhibitor Wilson and Kapoor (Wilson & Kapoor, 1993) achieved almost complete inhibition of PGI_2_ and PGE_2_ efflux evoked by rhythmic forearm contractions at low and moderate intensity: they concluded PGs were responsible for 10%–20% of the hyperemia that occurred in the relaxation phases of rhythmic contractions. Even the incomplete COX inhibition we achieved indicated that in young men, PGs are responsible for 24%–32% of *postcontraction* hyperemia following rhythmic *and* isometric contractions at moderate intensity. This agrees with estimates made from the effects of COX inhibitors on postcontraction hyperemia of moderate‐high intensity exercise (Cowley et al., 1985; Duffy et al., 1999; Kilbom & Wennmalm, 1976; Win & Marshall, 2005). But, our results also suggest PGs are responsible for at least 17%–21% of postcontraction hyperemia in recreationally active older men following rhythmic or isometric contractions at 60% MVC. Clearly, this contrasts with the proposal that the contribution of PGs to exercise hyperemia is lost with aging (Schrage et al., 2007). However, our finding that the percentage reduction in postcontraction hyperemia caused by COX inhibition was smaller in older, than young men agrees with evidence that the *action* of PGs is blunted with aging ( Nicholson et al., 2009).

### 4.3 Effects of 40% O_2_

In both young and older men, 40% O_2_ caused similar attenuation as aspirin of both PGI_2_ and PGE_2_ efflux, and the postcontraction hyperemia associated with rhythmic *and* isometric contractions. Moreover, 40% O_2_ and aspirin applied together had no greater effect on PG efflux, or the vasodilator responses. We previously showed in young men, that when breathing 40% O_2_ was restricted to the period of isometric contraction, it attenuated postcontraction hyperemia, but when given immediately after contraction, it had no such effect (Fordy & Marshall, 2012). Thus, we can now argue it is the *release,* rather than the action of PGI_2_ and PGE_2_ that allows PGs to make an O_2_‐dependent contribution to postcontraction hyperemia during both types of contraction, even though FBF and O_2_ delivery are more restricted during isometric contractions (Kagaya & Homma, 1997; Van Beekvelt et al., 2001). Importantly, our results indicate that PG release is just as O_2_‐dependent in recreationally active older men, as in young men.

Breathing 40% O_2_ raises arterial PO_2_ from ~90 to ~240 mmHg (Fordy & Marshall, 2012), and must have considerably steepened the O_2_ gradients within muscle. To be specific, with air breathing, PvO_2_ falls from ~40 mmHg at rest to 30–35 mmHg during submaximal exercise ( Dufour et al., 2010), whereas intracellular PO_2_ in muscle fibers falls from ~20–35 mmHg to ~3 mmHg during contractions at all intensities ≥50% maximum workload (Richardson, Newcomer, & Noyszewski, 2001). In this study, 40% O_2_ had no effect on PvO_2_ at rest, but alleviated the fall in PvO_2_ at the end of both rhythmic and isometric contractions, as reported by others (Dufour et al., 2010). Hyperoxia also raises muscle intracellular PO_2_ (Richardson, Noyszewski, Leigh, Wagner, 1998_)._ Thus, at rest, additional O_2_ must have diffused to the muscle fibers before reaching the veins, whereas during both types of contraction, O_2_ delivery must have exceeded O_2_ extraction such that the immediate postcontraction fall in PvO_2_ was prevented. Accordingly, 40% O_2_ must have raised the PO_2_ gradient along the vascular pathway and from vasculature to muscle fibers. During recovery, PvO_2_ increased above resting values as reported by others (Van Beekvelt et al., 2001) and 40% O_2_ exaggerated this. Thus, O_2_ delivery was greater than O_2_ extraction and 40% O_2_ exaggerated the disparity even though postcontraction hyperemia was attenuated by 40% O_2_.

Presumably 40% O_2_ increased arterial PO_2_ to a similar extent in older, as in young men and affected the PO_2_ gradients in a similar way. However, 40% O_2_ did not affect the fall in PvO_2_ in older men at the end of rhythmic, or isometric contractions. Moreover, PvO_2_ did not rise above resting values during recovery from either type of contraction, whereas 40% O_2_ raised PvO_2_ only at the 3rd min following isometric contraction. Thus, even though 40% O_2_ must have raised PO_2_ at least part way along the vascular pathway toward muscle fibers, additional O_2_ was extracted during both types of contractions and in recovery. This agrees with evidence that in recreationally active older men, O_2_ extraction normally reaches maximum in submaximal exercise (Proctor et al., 2003), and suggests 40% O_2_ can improve O_2_ extraction not only during contraction but also in recovery, even though 40% O_2_ attenuates postcontraction hyperemia.

### 4.4 Locations of PG release

PGI_2_ is the major PG released by the endothelium, the expression of PGI_2_ synthase being ~100‐fold greater than PGE_2_ synthase (Félétou, Huang, & Vanhoutte, 2011). On the other hand, PGE_2_ is the dominant PG released by skeletal muscle fibers (McLennan & Macdonald, 1991; Trappe & Liu, 2013). Endothelial PGI_2_ synthesis is maintained with aging (Félétou et al., 2011), whereas PGE_2_ synthesis in muscle during exercise *increases* with age (Trappe & Liu, 2013), consistent with our finding of a trend for PGE_2_ efflux following isometric contraction to be greater in older, than in young men. Thus, it is reasonable to conclude that in recreationally active young and older men, the PGI_2_ and PGE_2_ in venous efflux following isometric *and* rhythmic contractions originated mainly from endothelium and muscle fibers, respectively.

In resting muscle, perivascular PO_2_ falls from ~50 mmHg around larger arterioles, to 28–30 mmHg around capillaries and postcapillary venules and ~33 mmHg around larger venules (Lash & Bohlen, 1987). During submaximal muscle contractions, periarteriolar PO_2_ falls only transiently by ~10–20 mmHg, returning to resting values as the arterioles dilate, whereas pericapillary and perivenular PO_2_ falls by ~50% with little recovery until contraction ceases (Lash & Bohlen, 1987). Thus, capillaries and postcapillary venules are the most likely sites for a fall in endothelial PO_2_ to act as a stimulus for synthesis and release of PGI_2_ into venous efflux and from their extraluminal surfaces into the interstitium (Hester & Hammer, 2002; Lash & Bohlen, 1987). This proposal is consistent with evidence that endothelial cells release ~10‐fold more PGI_2_ than PGE_2_ at PO_2_ levels comparable to those reached during contraction (Michiels et al., 1993) and with venules releasing PGs during muscle contraction which dilate arterioles (Hester & Hammer, 2002; McKay, Gardner, Boyd, & Hester, 1998). On the other hand, arterial occlusion of forearm, which profoundly reduces muscle intracellular PO_2_ (Richardson et al., 2001) led to a threefold increase in efflux of PGE‐like substance (Kilbom & Wennmalm, 1976). Furthermore, contractions at ≥50% MVC reduce muscle intracellular PO_2_ (Richardson et al., 2001) and cause PGE_2_ release (Trappe & Liu, 2013), whereas 70% MVC contraction during arterial occlusion exacerbated PGE_2_ efflux (Symons et al., 1991). Thus, it seems likely the fall in muscle intracellular PO_2_ that occurs during contractions modulates, or triggers PGE_2_ release from muscle fibers.

Set against this background, it seems reasonable to propose that in both young and older men 40% O_2_ limits the fall in local PO_2_ sufficiently to attenuate release of PGI_2_ from capillary and venular endothelium and PGE_2_ from skeletal muscle fibers such that interstitial PG levels are reduced and postcontraction dilatation and hyperemia are attenuated.

### 4.5 Interdependent influences

ATP release from contracting muscle fibers is dependent on acidosis and lactic acid efflux (Tu, Lu, Cai, & Ballard, 2012), whereas ATP metabolism to adenosine is facilitated when PO_2_ falls (Marshall, 2007). Since 40% O_2_ did not affect lactate or H^+^ efflux in young or older men, it seems unlikely this ATP release was affected by contractions at 60% MVC. But, adenosine and ATP are also released from endothelial cells by a fall in PO_2_ (Edmunds, Moncada, & Marshall, 2003; To, Kumar, & Marshall, 2015), whereas erythrocytes release ATP when hemoglobin off‐loads O_2_ (Ellsworth & Sprague, 2012) and both adenosine and ATP release PGI_2_ from endothelial cells (Nyberg et al., 2013; Nyberg, Mortensen, Thaning, Saltin, & Hellsten, 2010; Ray, Abbas, Coney, & Marshall, 2002). Thus, 40% O_2_ may have attenuated exercise hyperemia by reducing the release and actions of ATP and adenosine whose contributions are partly mediated by PGI_2_ (Marshall & Ray, 2012; Nyberg et al., 2010, 2013).

### 4.6 Experimental considerations

We always tested rhythmic and isometric contractions in that order rather than randomizing, so as to avoid variability caused by fatigue from one type of contraction affecting the response to the other. An “order effect” seems unlikely to have influenced our results for young and older subjects were able to maintain rhythmic contractions at 60% MVC for 3 min and FBF had fully recovered before isometric handgrip. Assays of arterial PGs would have allowed more accurate assessment of PG efflux. However, forearm exercise at moderate intensity did not previously increase arterial PGI_2_, or PGE_2_ (Wilson & Kapoor, 1993). Thus, it is unlikely arterial assays would have changed our conclusions.

Intra‐arterial infusion of COX inhibitor might have achieved more complete COX blockade than one oral dose (Wilson & Kapoor, 1993) and allowed better assessment of the PG contribution to postcontraction hyperemia. However, we avoided using O_2_ at concentrations >40% to more rigorously test the O_2_‐dependency of PG release, or hyperemia because high O_2_ concentrations generate reactive oxygen species, which attenuate endothelium‐dependent dilatation (Rousseau, Tesselaar, Henricson, & Sjöberg, 2010) and may blunt exercise hyperemia. Our evidence indicates 40% O_2_ does not have this effect (Caruana & Marshall, 2015; Marshall, Junejo, D'Souza, & Ray, 2015).

Although we recorded *post*contraction hyperemia following isometric contraction, we probably captured the majority of the hyperemia, for continuous Doppler recordings showed FBF increased *less* during isometric contraction at 60% MVC than 30% MVC, with a much larger postcontraction hyperemia (McNeil et al., 2015). Thus, the attenuating effects of 40% O_2_ and COX inhibition on peak (postcontraction) hyperemia reported in this study probably reflected majority of the PG contribution. During *rhythmic* contractions, FBF averaged over contraction and relaxation phases increased more at 75% than 25% MVC; with a dramatic increase in postexercise FBF only at 75 MVC (Van Beekvelt et al., 2001). But, the peak *postexercise* FBF equalled FBF recorded in the relaxation phases *between* rhythmic contractions (Van Beekvelt et al., 2001). Thus, it is likely the FBF we recorded immediately *post*exercise for rhythmic contractions at 60% MVC was comparable to FBF during the relaxation phases. Since PG efflux increased on cessation of rhythmic contractions at 60% MVC, it seems reasonable to propose PGs contributed to the increases in FBF *during* the contraction period as reported by others for moderate rhythmic contractions (Wilson & Kapoor, 1993). Continuous recordings of FBF during rhythmic contractions at 60% MVC will be required to assess the time course and magnitude of this PG contribution.

### 4.7 Perspectives

Postcontraction hyperemia not only allows wash out of vasodilator mediators generated during contraction, but it restores creatine phosphate (PCr) at a rate determined by O_2_ delivery, O_2_ diffusion, and capacity for oxidative phosphorylation (Haseler, Lin, & Richardson, 2004; Kemps Hareld, Prompers Jeanine, & Wessels, 2009; Layec, Haseler, & Richardson, 2013). Thus, blunted postcontraction hyperemia in aging and cardiovascular disease contribute to slower PCr recovery rates and may limit the ability to perform repetitive daily activities (Haseler et al., 2004; Kemps Hareld et al., 2009; Layec et al., 2013). The present findings indicate that use of proprietary COX inhibitors might similarly slow PCr recovery kinetics and hasten fatigue, particularly in older people. On the other hand, breathing 40% O_2_ during exercise may avoid the deleterious effects of COX inhibition while facilitating the benefits. For, raised PO_2_ in muscle during exercise would inhibit PGI_2_ and PGE_2_ synthesis, but allow increased O_2_ extraction during recovery at least in older men (Figure 3) despite the attenuated postcontraction hyperemia. Certainly, even in young men, 40% O_2_ given selectively during recovery from a maximal fatiguing forearm contraction improved performance in a second contraction undertaken a few minutes later (Fordy & Marshall, 2012).

### 4.8 Conclusions

Against a background of controversy over whether PGs are necessary for full expression of exercise hyperemia, we have shown that isometric and rhythmic contractions at moderate intensity (60% MVC) caused substantial release of PGI_2_ and PGE_2_ from forearm of young *and* older men and that COX inhibition attenuated postcontraction hyperemia for both types of contraction by at least 20% in both young and older men. Furthermore, in young and older men, the release of PGI_2_ and PGE_2_ and contribution to postcontraction hyperemia were similarly attenuated by breathing 40% O_2_, whereas combined 40% O_2_ and COX inhibition had no greater effect. Thus, we propose the release of PGs during moderate intensity (60% MVC) isometric and rhythmic contractions is O_2_‐dependent in both young *and* older men.

## CONFLICT OF INTERESTS

The authors have no competing interests to declare.

## AUTHOR CONTRIBUTIONS

JMM conceptualized the study; RTJ, CJR, and JMM designed the work; RTJ performed acquisition and data analysis; RTJ, CJR, and JMM interpreted the data; RTJ drafted the manuscript, which was critically revised by CJR and JMM. All authors approved the final manuscript.

## Supporting information



Figure S1

Table S1–S3
