# Supplementary figures and images for: Prostaglandin contribution to postexercise hyperemia is dependent on tissue oxygenation during rhythmic and isometric contractions
Source: Physiol Rep. 2020 Jun 19;8(12):e14471. doi: 10.14814/phy2.14471 (PMC7305242; doi:10.14814/phy2.14471)

(a) Rhythmic Handgrip

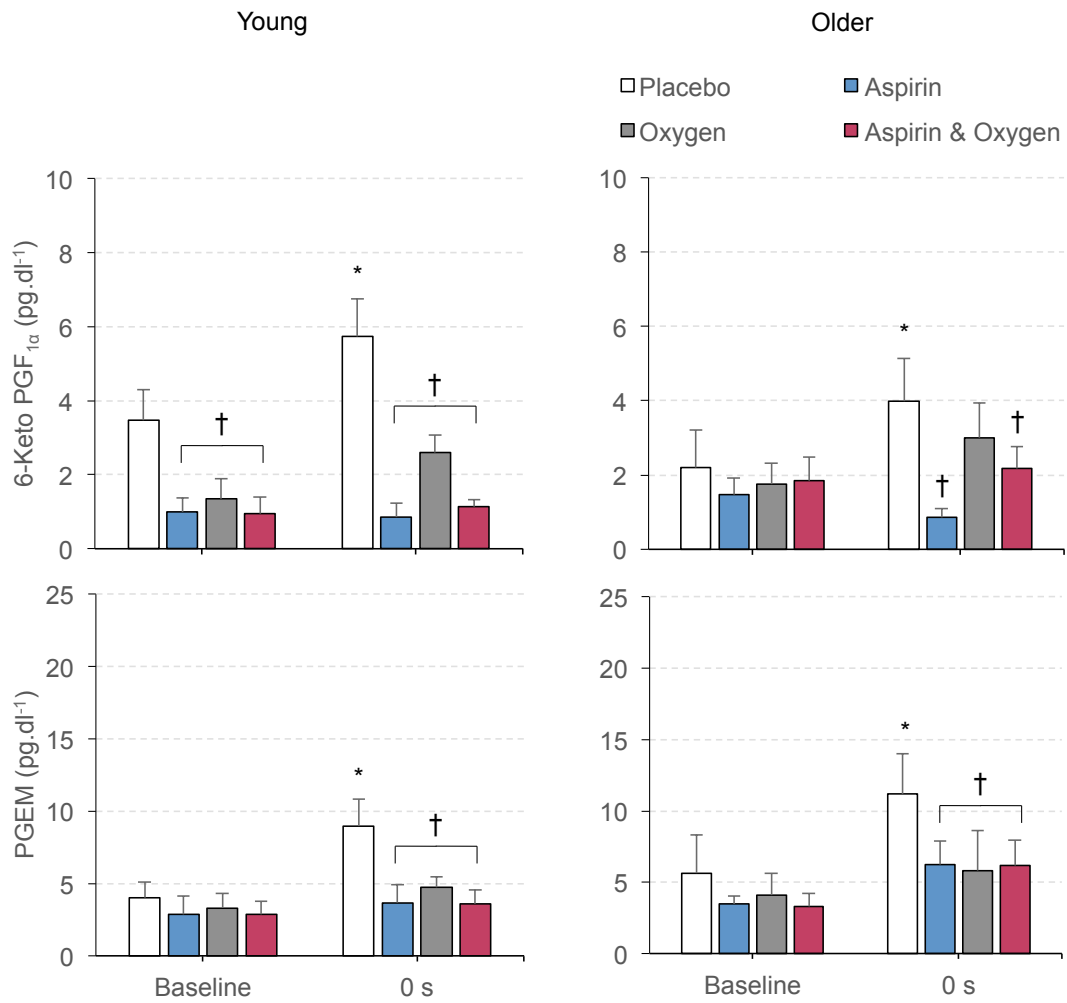

(b) Isometric Handgrip

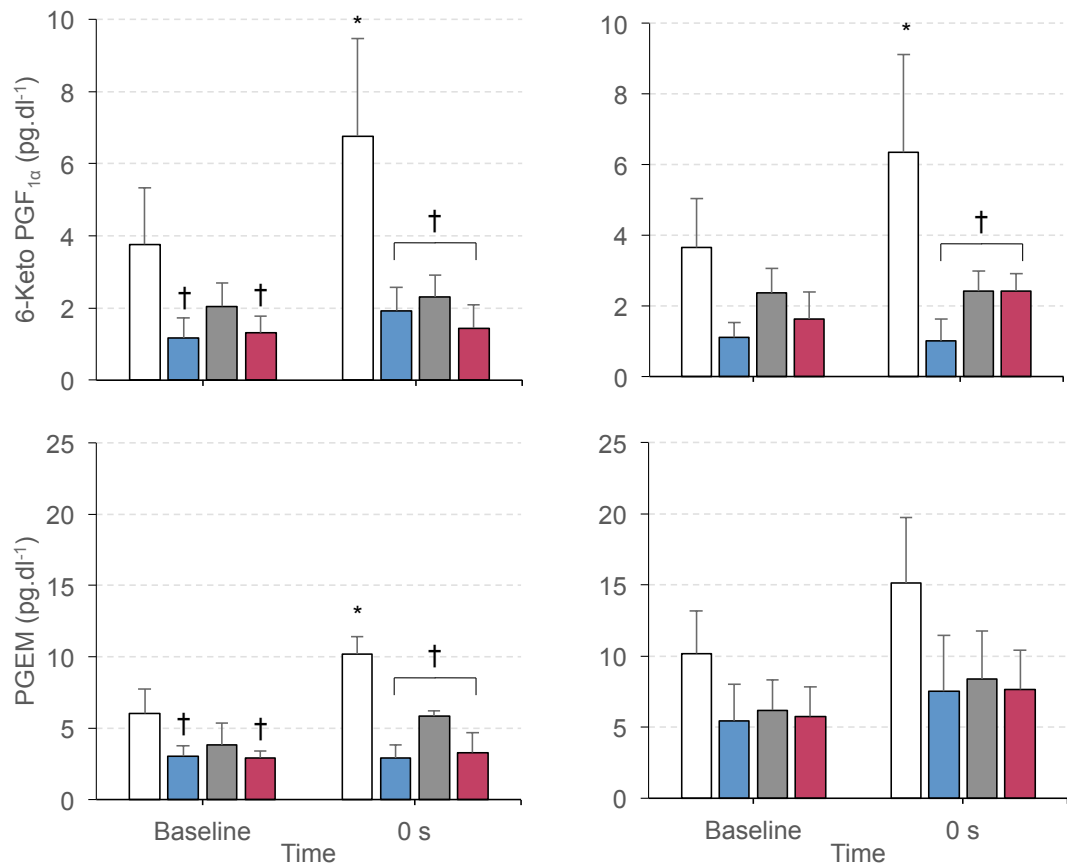

Supplement: Supplementary file 1 — Figure S1 [file PHY2-8-e14471-s001.pdf]
