# Supplementary material for: Prostaglandin contribution to postexercise hyperemia is dependent on tissue oxygenation during rhythmic and isometric contractions
Source: Physiol Rep. 2020 Jun 19;8(12):e14471. doi: 10.14814/phy2.14471 (PMC7305242; doi:10.14814/phy2.14471)
Supplement: Supplementary file 2 — Table S1–S3 [file PHY2-8-e14471-s002.docx]

**SUPPLEMENTARY MATERIALS**

**Supplementary Figure 1: Venous concentrations 6-Keto PGF_1α_ and PGEM in young men and older men during rhythmic (a) and isometric (b) contractions under control conditions (placebo), after Aspirin, during 40% O_2_, and during combined Aspirin+40%O_2_.** Values are mean ± SEM . *: P < 0.05 vs. respective baselines, †: P< 0.05 for all treatments vs placebo.

**Supplementary Table 1. Venous metabolites in young and older men before, on cessation and at intervals after rhythmic contractions after Placebo, after Aspirin, during 40% O_2_ and during combined Aspirin and 40% O_2_ (A+40%O_2_).** BL: baseline; PvO_2_: venous O_2_; PvCO_2_: venous CO_2_; K^+^: Potassium; pH: H^+^ ion concentration, *: values significantly different from respective baseline (BL), †: treatment effect (vs. placebo), ‡: Age effect (vs. young); P < 0.05. All values are given as mean ± SE.

**Supplementary Table 2. Venous metabolites in young and older men before, on cessation and at intervals after isometric contractions after Placebo, after Aspirin, during 40% O_2_ and during combined Aspirin and 40% O_2_ (A+40%O_2_).** BL: baseline; PvO_2_: venous O_2_; PvCO_2_: venous CO_2_; K^+^: Potassium; pH: H^+^ ion concentration, *: values significantly different from respective baseline (BL), †: treatment effect (vs. placebo), ‡: Age effect (vs. young); P < 0.05. All values are given as mean ± SE.

**Supplementary Table 3:** **Percentage changes in exercise hyperaemia and efflux of PG metabolites following rhythmic and isometric contractions in Young and Older men**. Values given as mean ± SEM. All values are taken from 6 young and 6 older men. P values for comparisons between conditions and between young and older men are shown in Figure 5.

|  |  | **Young** | | **Post-Exercise** | | | | | | | | | | **Older** | **Post-Exercise** | | | | | | |
| --- | --- | --- | --- | --- | --- | --- | --- | --- | --- | --- | --- | --- | --- | --- | --- | --- | --- | --- | --- | --- | --- |
| PvO_2_  mmHg |  | **BL** | | **0 min** | **3 min** | | | **5 min** | | | **7 min** | |  | **BL** | **0 min** | **3 min** | **5 min** | **7 min** | |  | |
|  | Placebo | 44±5 | | 34±3* | 52±3 | | | 51±3 | | | 51± 3 | | *0.02* | 43±5 | 30±3* | 51±2 | 48±4 | 46±4 | | *< 0.01* | |
|  | Aspirin | 42 ±3 | | 32±2* | 51 3 | | | 51±2 | | | 51±3 | | *< 0.01* | 52±3 | 30±1* | 51±2 | 52±2 | 53±3 | | *< 0.01* | |
|  | 40% O_2_† | 44±4 | | 39±3 | 58±4* | | | 60±4* | | | 58±4* | | *< 0.01* | 53±6 | 31±2* | 53±2 | 54±3 | 55±4 | | *< 0.01* | |
|  | A+O_2_† | 41±3 | | 35±3 | 57±2* | | | 58±2* | | | 57±2* | | *< 0.01* | 53±4 | 28±2* | 50±3 | 51±4 | 49±4 | | *< 0.01* | |
|  |  | † Treatment P < 0.05; Time*Treatment P: 0.12 | | | | | | | | | | | | Treatment: 0.91; Time*Treatment: 0.71; Age*Treatment: 0.39 | | | | | | | |
| PvCO_2_  mmHg | Placebo | 53±2 | | 99±6* | 63±2 | | | 54±2 | | | 52±2 | | *< 0.01* | 47±2 | 82±5* | 55±1* | 50±1 | 48±2 | | *< 0.01* | |
|  | Aspirin | 50±2 | | 88±6* | 58±1 | | | 55±1 | | | 50±1 | | *< 0.01* | 45±1 | 80±8* | 51±2* | 46±1 | 45±1 | | *< 0.01* | |
|  | 40% O_2_ | 52±3 | | 93±5* | 60±1 | | | 51±1 | | | 52±1 | | *< 0.01* | 46±1 | 91±6* | 56±1* | 51±1 | 50±1 | | *< 0.01* | |
|  | A+O_2_ | 51±3 | | 94±10* | 60±2 | | | 54±1 | | | 53±2 | | *< 0.01* | 49±2 | 84±6* | 59±2* | 53±3 | 51±3 | | *< 0.01* | |
|  |  | Treatment: P = 0.61; Time*Treatment P = 0.96 | | | | | | | | | | | | Treatment:0.26; Time*Treatment:0.87; Age*treatment:0.30 | | | | | | | |
| K^+^  mmol.L^-1^ | Placebo | 4.7±0.1 | | 5.3±0.2* | 4.6±0.1 | | | 5.0±0.3 | | | 5.0±0.3 | | *0.02* | 4.8±0.2 | 6.3±0.6* | 4.7±0.2 | 4.7±0.3 | 4.7±0.2 | | *< 0.01* | |
|  | Aspirin | 4.6±0.1 | | 5.3±0.2* | 4.4±0.1 | | | 4.4±0.1 | | | 4.6±0.1 | | *< 0.01* | 5.0±0.2 | 5.7±0.2* | 5.0 ±0.1 | 4.7±0.1 | 4.9±0.1 | | *< 0.01* | |
|  | 40% O_2_ | 4.7±0.1 | | 5.1±0.3* | 4.5±0.1 | | | 4.4±0.1 | | | 4.7±0.2 | | *0.03* | 4.9±0.1 | 5.8±0.2* | 4.7±0.1 | 4.9±0.1 | 4.9±0.2 | | *< 0.01* | |
|  | A+O_2_ | 4.6±0.1 | | 5.4±0.1* | 4.5±0.1 | | | 4.6±0.1 | | | 4.6±0.1 | | *< 0.01* | 4.8±0.2 | 5.8±0.2* | 4.8±0.2 | 4.9±0.2 | 5.0±0.2 | | *< 0.01* | |
|  |  | Treatment P = 0.16; Time*Treatment P = 0.20 | | | | | | | | | | | | Treatment:0.91; Time*Treatment:0.06; Age*treatment:0.42 | | | | | | | |
| pH | Placebo‡ | 7.4±0.01 | | 7.2±0.01* | 7.2±0.01* | | | 7.3±0.01* | | | 7.3±0.01* | | *< 0.01* | 7.4±0.01 | 7.2±0.02* | 7.3±0.01* | 7.3±0.01 | 7.4±0.02 | | *< 0.01* | |
|  | Aspirin‡ | 7.4±0.01 | | 7.2±0.02* | 7.3±0.01* | | | 7.3±0.02* | | | 7.3±0.01* | | *< 0.01* | 7.4±0.01 | 7.2±0.03* | 7.3±0.02* | 7.4±0.01 | 7.4±0.01 | | *< 0.01* | |
|  | 40% O_2_ | 7.4±0.01 | | 7.2±0.02* | 7.3±0.01* | | | 7.3±0.01 | | | 7.3±0.01 | | *< 0.01* | 7.4±0.00 | 7.2±0.01* | 7.3±0.01* | 7.3±0.01* | 7.3±0.01* | | *< 0.01* | |
|  | A+O_2_ | 7.4±0.02 | | 7.2±0.02* | 7.3±0.01* | | | 7.3±0.01* | | | 7.3±0.01 | | *< 0.01* | 7.4±0.01 | 7.2±0.02* | 7.3±0.02* | 7.3±0.01* | 7.3±0.02* | | *< 0.01* | |
|  |  | Treatment P = 0.53; Time*Treatment P = 0.78 | | | | | | | | | | | | Treatment:0.30; Time*Treatment:0.54; ‡ Age*Treatment < 0.05 | | | | | | | |
| Lactate mmol.L^-1^ | Placebo‡ | 1.3±0.1 | 5.2±0.6* | | | | 5.0±0.4* | | 4.5±0.4* | | 4.0±0.5* | *< 0.01* | | 1.3±0.2 | 4.2±0.4* | 3.9±0.3* | 3.3±0.4* | | 3.0±0.4* | | *< 0.01* |
|  | Aspirin‡ | 1.4±0.2 | 4.7±0.4* | | | | 4.5±0.4* | | 4.1±0.5* | | 3.7±0.4* | *< 0.01* | | 1.3±0.1 | 3.7±0.4* | 3.4±0.3* | 3.0±0.3* | | 2.4±0.2* | | *< 0.01* |
|  | 40% O_2_‡ | 1.3±0.2 | 5.0±0.6* | | | | 5.1±0.5* | | 4.5±0.5* | | 3.8±0.4* | *< 0.01* | | 1.3±0.1 | 4.3±0.3* | 3.6±0.3* | 3.4±0.3* | | 3.0±0.3* | | *< 0.01* |
|  | A+O_2_‡ | 1.3±0.1 | 5.1±0.4* | | | 5.2±0.3* | | | | 4.8±0.4* | 4.2±0.4* | *< 0.01* | | 1.3±0.1 | 3.7±0.4* | 3.7±0.4* | 3.3±0.2* | | 3.0±0.3* | | *< 0.01* |
|  |  | Treatment P = 0.86; Time*Treatment P = 0.97 | | | | | | | | | | | | Treatment:0.77; Time*Treatment:0.86; ‡ Age*Treatment < 0.05 | | | | | | | |

|  |  | **Young** | | **Post-Exercise** | | | | | | | | | | **Older** | **Post-Exercise** | | | | | | |
| --- | --- | --- | --- | --- | --- | --- | --- | --- | --- | --- | --- | --- | --- | --- | --- | --- | --- | --- | --- | --- | --- |
| PvO_2_  mmHg |  | **BL** | | **0 min** | **3 min** | | | **5 min** | | | **7 min** | |  | **BL** | **0 min** | **3 min** | **5 min** | **7 min** | |  | |
|  | Placebo | 47±5 | | 38±5* | 58±3* | | | 61±5* | | | 59±3* | | *< 0.01* | 50±3 | 36±4* | 54±3 | 62±7 | 53±5 | | *< 0.01* | |
|  | Aspirin | 45 ±5 | | 38±2* | 55±2* | | | 57±2* | | | 60±4* | | *< 0.01* | 52±2 | 34±2* | 59±2* | 56±2 | 56±3 | | *< 0.01* | |
|  | 40% O_2_† | 49±3 | | 46±4 | 71±4* | | | 69±4* | | | 72±12* | | *< 0.01* | 49±5 | 37±3* | 60±3* | 59±5 | 56±5 | | *< 0.01* | |
|  | A+O_2_† | 52±5 | | 39±2* | 65±3* | | | 63±3* | | | 75±12* | | *0.01* | 48±5 | 33±1* | 61±3 | 58±4 | 55±5 | | *< 0.01* | |
|  |  | † Treatment P < 0.05; Time*Treatment P: 0.83 | | | | | | | | | | | | Treatment: 0.99; Time*Treatment: 0.61; Age*Treatment: 0.06 | | | | | | | |
| PvCO_2_  mmHg | Placebo | 48±2 | | 96±7* | 56±3 | | | 50±2 | | | 48±2 | | *< 0.01* | 44±1 | 75±10* | 49±2 | 45±2 | 47±2 | | *< 0.01* | |
|  | Aspirin | 49±1 | | 90±7* | 55±2 | | | 50±2 | | | 48±1 | | *< 0.01* | 42±1 | 77±8* | 48±1 | 46±1 | 44±1 | | *< 0.01* | |
|  | 40% O_2_ | 48±1 | | 87±8* | 56±2 | | | 52±2 | | | 49±1 | | *< 0.01* | 47±1 | 67±4* | 54±2 | 48±2 | 46±1 | | *< 0.01* | |
|  | A+O_2_ | 46±3 | | 88±10* | 52±2 | | | 51±2 | | | 47±1 | | *< 0.01* | 47±2 | 74±6* | 53±2 | 52±1 | 46±1 | | *< 0.01* | |
|  |  | Treatment: P = 0.77; Time*Treatment P = 0.99 | | | | | | | | | | | | Treatment:0.73; Time*Treatment:0.58; Age*treatment:0.41 | | | | | | | |
| K^+^  mmol.L^-1^ | Placebo | 4.7±0.1 | | 6.3±0.4* | 5.2±0.4 | | | 4.9±0.3 | | | 4.9±0.1 | | *< 0.01* | 4.7±0.2 | 5.3±0.3* | 5.0±0.4 | 5.1±0.3 | 5.2±0.3 | | *< 0.05* | |
|  | Aspirin | 4.8±0.1 | | 5.7±0.2* | 4.9±0.1 | | | 4.7±0.1 | | | 4.8±0.1 | | *< 0.01* | 5.1±0.1 | 5.7±0.2* | 5.0 ±0.1 | 5.1±0.2 | 5.0±0.2 | | *0.02* | |
|  | 40% O_2_ | 4.9±0.1 | | 5.8±0.3* | 5.1±0.4 | | | 4.8±0.3 | | | 5.2±0.4 | | *0.04* | 4.8±0.2 | 5.7±0.3* | 4.9±0.2 | 4.9±0.1 | 4.8±0.1 | | *< 0.01* | |
|  | A+O_2_ | 4.6±0.2 | | 6.0±0.3* | 4.7±0.1 | | | 5.2±0.4 | | | 5.0±0.2 | | *0.04* | 4.8±0.2 | 5.4±0.2* | 5.0±0.2 | 5.2±0.3 | 5.1±0.2 | | *< 0.01* | |
|  |  | Treatment P = 0.79; Time*Treatment P = 0.80 | | | | | | | | | | | | Treatment:0.98; Time*Treatment:0.87; Age*treatment:0.38 | | | | | | | |
| pH | Placebo‡ | 7.4±0.01 | | 7.2±0.02* | 7.3±0.02* | | | 7.3±0.01 | | | 7.3±0.02 | | *< 0.01* | 7.4±0.01 | 7.2±0.03* | 7.3±0.01* | 7.4±0.01 | 7.4±0.01 | | *< 0.01* | |
|  | Aspirin‡ | 7.4±0.01 | | 7.2±0.03* | 7.3±0.02* | | | 7.3±0.02 | | | 7.3±0.02 | | *< 0.01* | 7.4±0.01 | 7.2±0.04* | 7.3±0.01* | 7.4±0.01 | 7.4±0.01 | | *< 0.01* | |
|  | 40% O_2_ | 7.4±0.01 | | 7.2±0.03* | 7.3±0.01* | | | 7.3±0.02* | | | 7.3±0.01 | | *< 0.01* | 7.4±0.01 | 7.3±0.01* | 7.3±0.01* | 7.3±0.01 | 7.4±0.01 | | *< 0.01* | |
|  | A+O_2_ | 7.4±0.02 | | 7.2±0.03* | 7.3±0.02* | | | 7.3±0.02* | | | 7.3±0.02 | | *< 0.01* | 7.4±0.01 | 7.2±0.03* | 7.3±0.01* | 7.3±0.01 | 7.4±0.01 | | *< 0.01* | |
|  |  | Treatment P = 0.53; Time*Treatment P = 0.78 | | | | | | | | | | | | Treatment:0.43; Time*Treatment:0.70; ‡ Age*Treatment < 0.05 | | | | | | | |
| Lactate mmol.L^-1^ | Placebo‡ | 1.4±0.1 | 5.7±0.7* | | | | 4.8±0.3* | | 4.2±0.4* | | 3.6±0.4* | *< 0.01* | | 1.4±0.2 | 3.4±0.5* | 3.0±0.2* | 2.9±0.3* | | 2.7±0.3* | | *< 0.01* |
|  | Aspirin‡ | 1.4±0.1 | 5.7±0.6* | | | | 4.7±0.6* | | 4.3±0.6* | | 3.6±0.4* | *< 0.01* | | 1.3±0.1 | 3.7±0.5* | 3.1±0.3* | 2.8±0.2* | | 2.3±0.2* | | *< 0.01* |
|  | 40% O_2_‡ | 1.6±0.2 | 5.8±0.6* | | | | 5.6±0.6* | | 5.0±0.5* | | 4.3±0.5* | *< 0.01* | | 1.3±0.1 | 3.4±0.3* | 3.3±0.2* | 3.0±0.3* | | 2.6±0.2* | | *< 0.01* |
|  | A+O_2_‡ | 1.6±0.2 | 5.4±0.5* | | | 5.1±0.5* | | | | 4.8±0.6* | 3.8±0.3* | *< 0.01* | | 1.4±0.1 | 3.6±0.5* | 3.3±0.2* | 3.3±0.3* | | 2.8±0.2* | | *< 0.01* |
|  |  | Treatment P = 0.69; Time*Treatment P = 0.98 | | | | | | | | | | | | Treatment:0.84; Time*Treatment:0.97; ‡ Age*Treatment < 0.05 | | | | | | | |

|  |  |  | **Aspirin** | **40% O_2_** | **Aspirin + 40% O_2_** |
| --- | --- | --- | --- | --- | --- |
| FBF | Young | Rhythmic | 23.5 ± 4.7 | 23.5 ± 4.9 | 24.4 ± 6.0 |
|  |  | Isometric | 28.3 ± 5.9 | 30.2 ± 3.6 | 30.9 ± 4.4 |
|  | Older | Rhythmic | 17.6 ± 3.4 | 16.2 ± 3.2 | 18.3 ± 2.1 |
|  |  | Isometric | 20.9 ± 2.6 | 22.5 ± 2.3 | 19.4 ± 3.0 |
| 6-Keto PGF_1α_ | Young | Rhythmic | 86.6 ± 4.5 | 74.5 ± 8.5 | 87.9 ± 3.5 |
|  |  | Isometric | 68.5 ± 12.4 | 70.7 ± 8.9 | 75.6 ± 8.1 |
|  | Older | Rhythmic | 79.5 ± 8.0 | 66.6 ± 9.5 | 70.0 ± 15.9 |
|  |  | Isometric | 85.3 ± 3.1 | 66.6 ± 7.9 | 73.7 ± 6.2 |
| PGEM | Young | Rhythmic | 62.7 ± 9.8 | 63.9 ± 10.0 | 62.5 ± 21.9 |
|  |  | Isometric | 74.1 ± 10.9 | 67.4 ± 9.2 | 78.5 ± 5.1 |
|  | Older | Rhythmic | 52.1 ± 14.0 | 55.0 ± 19.6 | 48.9 ± 14.0 |
|  |  | Isometric | 53.3 ± 11.6 | 66.4 ± 12.7 | 65.6 ± 11.3 |
